# Now and Next-Generation Sequencing Techniques: Future of Sequence Analysis Using Cloud Computing

**DOI:** 10.3389/fgene.2012.00280

**Published:** 2012-12-11

**Authors:** Radhe Shyam Thakur, Rajib Bandopadhyay, Bratati Chaudhary, Sourav Chatterjee

**Affiliations:** ^1^Department of Biotechnology, Birla Institute of TechnologyMesra, Ranchi, India; ^2^Center for Computational Natural Sciences and Bioinformatics, International Institute of Information TechnologyHyderabad, India

**Keywords:** next-generation sequencing, cloud computing, DNA cloud

## Abstract

Advances in the field of sequencing techniques have resulted in the greatly accelerated production of huge sequence datasets. This presents immediate challenges in database maintenance at datacenters. It provides additional computational challenges in data mining and sequence analysis. Together these represent a significant overburden on traditional stand-alone computer resources, and to reach effective conclusions quickly and efficiently, the virtualization of the resources and computation on a pay-as-you-go concept (together termed “cloud computing”) has recently appeared. The collective resources of the datacenter, including both hardware and software, can be available publicly, being then termed a public cloud, the resources being provided in a virtual mode to the clients who pay according to the resources they employ. Examples of public companies providing these resources include Amazon, Google, and Joyent. The computational workload is shifted to the provider, which also implements required hardware and software upgrades over time. A virtual environment is created in the cloud corresponding to the computational and data storage needs of the user via the internet. The task is then performed, the results transmitted to the user, and the environment finally deleted after all tasks are completed. In this discussion, we focus on the basics of cloud computing, and go on to analyze the prerequisites and overall working of clouds. Finally, the applications of cloud computing in biological systems, particularly in comparative genomics, genome informatics, and SNP detection are discussed with reference to traditional workflows.

## Introduction

The accumulation of DNA sequence information, comprising merely the order within a simple polymer of the four canonical bases (A, T, G, C), has suddenly exploded into the bioscientific universe, drawing comparisons to the Big Bang theory of the origin of the universe. The development of increasingly high-throughput sequencing techniques has revolutionized the DNA world, producing extensive DNA datasets housed at various locations around the world. Collectively, this information has taken the form of cloud, and may be accurately termed a “DNA cloud.” The explosion in DNA sequence accumulation can be traced to developments including pyrosequencing (Franca et al., 2002), nanopore sequencing (Branton et al., 2008; Ivanov et al., 2011), single molecule sequencing (SMS) technology using DNA polymerases (Nusbaum, 2009), non-optical sequencing based on detection of pH changes (Rothberg et al., 2011), and high-throughput short-read platforms such as the Illumina Miseq and Hiseq sequencers (Caporaso et al., 2012). Despite the development of computers consistently following Moore’s law in terms of processing speed, this aspect now lags the data storage and maintenance requirements for the large amount of DNA sequence data produced by high-throughput next-generation sequencing techniques (Shendure and Ji, 2008). To cope with this situation, techniques of parallel computation using virtual hardware, software, and working platform resources have appeared and collectively termed “cloud computing.”

Although the term cloud has been loosely employed as a metaphor for the internet, today the scenario has changed leading to a change in the definition of cloud computing which hearkens back to an earlier definition provided by John McCarthy in 1961, who said, “computation may someday be organized as public utility” (Speech given to MIT Centennial; Garfinkel, 1999). Douglas Parkhill, in his book entitled “The challenge of the computer utility” describes all the modern-day characteristics of cloud computing, which includes providing an elastic environment as a utility, which can be used in several forms by the public, private, and community with differing usage. Although controversy persists over how to properly define a cloud, Forrester’s definition (Harris, 2009) appears most appropriate: a cloud is a pool of abstracted, highly scalable, and managed computer infrastructure capable of hosting end-customer applications and billed by consumption.

In a broader sense, cloud computing can be defined as an elastic execution environment of resources, involving multiple stakeholders and providing a metered service at multiple granularities for a specified level of quality of service (Vermesan and Friess, 2011). Given that cloud computing is now emerging as a commercial reality, the following points appear to underpin the reason for its commercialization (Armbrust et al., 2009):

1. Data amount: Due to recent advancements in analytical techniques, huge amounts of data are generated per day in different fields of education and research. To maintain such large data sets and provide for their efficient processing, the concept of cloud computing offers practical advantages.
2. Technology support: The development of simple and secure online payment modes, the increases in internet and network speeds accompanying introduction of 2G and 3G services and advancements in data compression techniques, all favor implementation of cloud computing.
3. Cost: Advances in miniaturization technology have continuously reduced the overall cost of cloud computing, as compared to earlier technologies (such as Grid Computing, Distributed system, and Utility Computing) which previously were employed in large datacenters. Presently, Intel is designing a Single Chip Cloud Computer which integrates a cloud of computers into an integrated chip (Harris, 2009).

## Components of Computational Clouds and Their Structure

**Figure 1:**
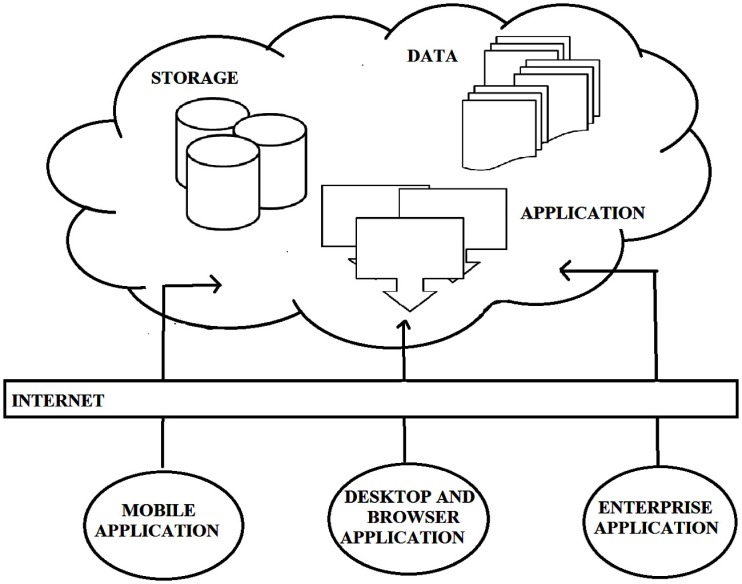
**The basic model of cloud computing along with components of cloud**. Unlike traditional beliefs internet is just the medium to provide services rather cloud [courtesy Torry Harris Business Solutions (THBS), copyright 2009 (www.thbs.com) with kind permission and slightly modified].

Historically, the definition of computational clouds has not been fixed, but has changed to accommodate developments in hardware and software; we fully expect its definition to change in adapting to future developments. At the present time, computational clouds comprise the application(s) used to extract information from raw data, the database storing all the information, and the physical storage system and servers. Computational clouds are configured to provide services to end-users (termed “clients”) via high speed internet connections. Cloud components and basic cloud computing models are illustrated in Figure 1:

## Types of Cloud

Based on the availability of the datacenter and the related applications to the clients, clouds are of the following three types (Rao and Rao, 2009):

### Public clouds

These are clouds owned and operated by third parties, aiming at individual client satisfaction by providing services at lower cost using a pay-as-you-go manner. An identical infrastructure pool is shared by all clients, operating with general constraints such as data security, and limited configuration to data and data variance. All the services are maintained by the cloud providers and may satisfy various needs as per demand. These services may be accessed from within the enterprise (by the user). These cloud services allow for a much greater size than may be possible within the enterprise using the cloud. Amazon’s Elastic Compute Cloud (EC2), IBM’s Blue Cloud, Sun Cloud, Google’s App Engine, and Windows Azure Services are some examples of the few public clouds.

### Private clouds

These are clouds that are owned and operated by an enterprise solely for its own use. Data security and control are generally stronger than in public clouds. NASA’s Nebula and Amazon’s virtual private cloud (VPN) are private clouds. Private clouds are organized into the following two types:

- On Premise Private Cloud: Clouds falling in this category maintain clouds within the data center of the organization. This provides strong control on data and its flow, and thus best suited for private enterprises requiring high security. They are also known as internally hosted clouds.
- Off Premise Private Cloud: This type of cloud shares data centers from different enterprises to form clouds. The security level may be a little less stringent, due to the fact that data centers are shared. This is best suited for enterprises who are not interested in sharing physical storage, but that wish not to compromise on security level of data. They are also known as externally hosted clouds.

### Hybrid clouds

These are combinations of both public and private clouds. The private cloud providers can use a third-party provider, either in partial or full manner, and provide the service to its enterprise. The augmentation of private and public clouds via hybrid clouds significantly reduces workloads.

## Models of Cloud Computing

The services provided under cloud computing can be grouped into the following three categories:

1. Software as a Service (SaaS): As this name suggests, software (the complete application) is served on demand to the clients. Multiple users are serviced using the single instance of software, without investing in servers and licenses. Only the provider has to pay, and, as a single instance, the running cost is much lower than on multiple servers.
2. Platform as a Service (PaaS): Here a working platform is provided as a service by encapsulating the required software and the working environment to the provider, with this platform then being used by the clients. Many platforms fall into this category, for example, Restricted Java 2 Enterprise Edition (J2EE), RUBY, Linux, Apache, MySql, PHP (LAMP), with APPERENDA to fulfill manageability and scalability requirements.
3. Infrastructure as a Service (Iaas): This service provides computing capabilities and basic storage over the network. Here networking equipments, data center space and servers are provided as standardized service. Most common are Amazon, Joyent, GoGrid, and Skytap.

## Service Providers

The cloud service is offered and maintained internationally and nationally by various companies. The clients access computational resources over the internet via vendors (for example Amazon EC2) in the form of some performance platform such as the Galaxy cloud (Afgan et al., 2011). International cloud providers (Table 1) specialize in different use-cases and hence offer specific services.

**Table 1:** **Name and address of the international cloud service provider, types of clouds, and their web link**.

Sl. No	Company	Address	Cloud offering	Web link
1	Amazon	Street address: 1200 12th Avenue south, Seattle, WA, USA	Elastic compute cloud	http://aws.amazon.com/ec2/
2	Bluelock	5303 Lakeview Parkway South Drive Indianapolis, IN, USA	Vcloud	http://www.bluelock.com/
3	CSC	3170, Fairview, Park drive, Falls church, VA, USA	Compute cloud, cloud lab	http://www.csc.com/cloud
4	Google	Daniel Ferguson, Miami, USA	App engine	http://www.googlecloudcomputing.net/
5	IBM	IBM corporation, White plains, NY, USA	Blue cloud, sun cloud	http://www.ibm.com/cloud-computing/us/en/
6	Joyent	Joyent, 345 San francisco, CA, USA	Smart machine	http://www.joyentcloud.com/
7	Microsoft	Microsoft Corporation, Redmond, WA, USA	Azure	http://www.microsoft.com/en-in/servercloud/readynow/default.aspx
8	Rackspace	Rackspace, 5000 Walzem, TX, USA	Openstack	http://www.rackspace.com/
9	Salesforce	The Landmark @ One Market suite, San francisco, CA, USA	Salescloud	http://www.salesforce.com/cloudcomputing/
10	THBS	Torry Harris business solutions, 536 Fayette, USA	N/A	http://www.thbs.com/

## Applications in Biological System

The application of bioinformatics tools and computational biology to results obtained from wet lab experiments provides a means to refine these results and their predictions. It is therefore of paramount importance in research and developmental studies to achieve reduced time consumption in computational activities, despite exponentially growing datasets. The application of cloud computing has provided a means to address this problem, and applications have been already described in neurosciences (Watson et al., 2008), biomedical informatics (Rosenthal et al., 2010), and bioimage informatics (Peng, 2008). In this section, we briefly discuss case-study uses of cloud computing in the following subfields of genome analysis: SNP detection, comparative genomics, genome informatics, and metagenomics.

### Genome analysis and SNP detection

In the wet lab, SNP detection and validation typically requires large numbers of PCR runs occupying many months of time. For example, for analysis of a mere 48 contigs of bread wheat, 1260 PCR runs needs to be performed just for SNP detection (Rustgi et al., 2009). The validation and assembly then required resequencing, which occupied even more time. The above process if implemented on cloud can result to provide following advantages.

Firstly, using bioinformatic approach, a single conventional computer required weeks of time to analyze a deep coverage human resequencing project and annotate the whole-genome (Rust et al., 2002). The cloud application (Hadoop, implementing MapReduce) whereas solved the same analysis problem in less than 3 h without compromising the accuracy rate (Schadt et al., 2010; Schatz et al., 2010).

**Figure 2:**
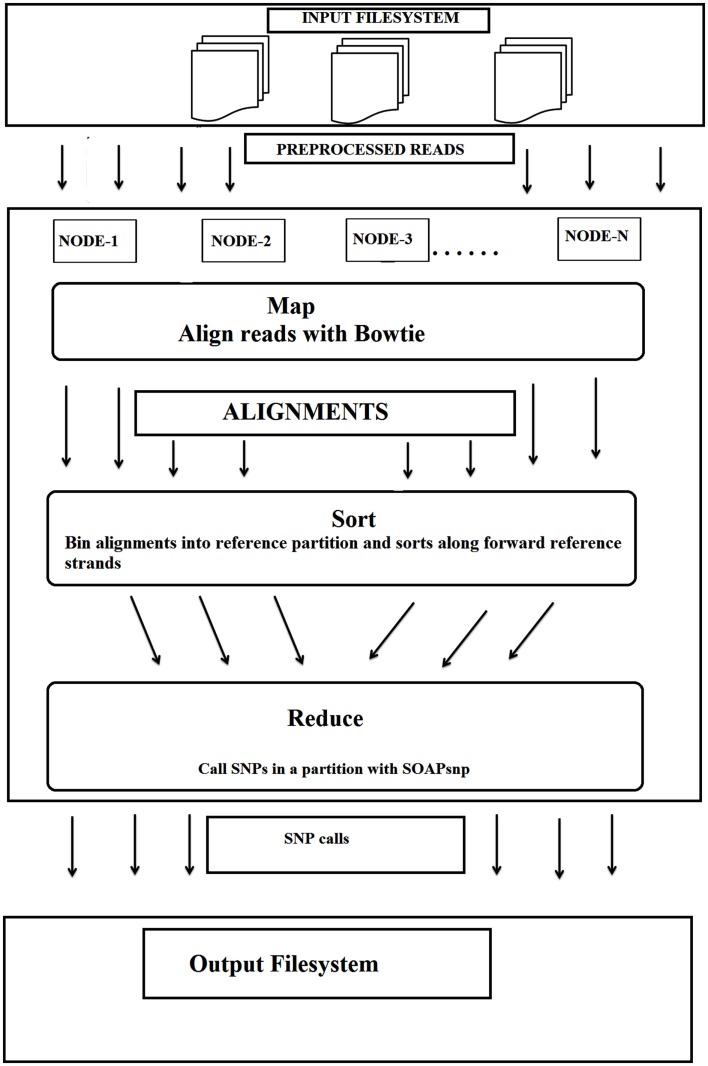
**The workflow for the detection of SNPs using cloud computing**. Bowtie and SOAPsnp were used in this workflow (adopted from Langmead et al., 2009 with kind permission and slightly modified).

Secondly, this particular cloud implementation used an efficient whole-genome genotyping tool, Crossbow, combining two software tools (Langmead et al., 2009), the sequence aligner “Bowtie” and the SNP caller “SOAPsnp.” This combination allowed rapid analysis of large sets of DNA sequences whilst maintaining an accuracy >98.9% with simulated datasets of individual chromosomes and >99.8% concordance with the Illumina 1 M BeadChip assay of a sequenced individual. This implementation also did not require extensive software engineering for parallel computation. The input file system was distributed over several nodes in cloud environment (MapReduce), with Bowtie being called for alignment, followed by SOAPsnp for SNP detection on every allotted server (Figure 2).

Cloud computing realized by MapReduce and Hadoop can be leveraged to efficiently parallelize existing serial implementations of sequence alignment and genotyping algorithms. This combination allows large datasets of DNA sequences to be analyzed rapidly without sacrificing accuracy or requiring extensive software engineering efforts to parallelize the computation (Langmead et al., 2009). Another cloud based pipeline CloudMap greatly simplifies the analysis of mutant genome sequences. It is available on the Galaxy web platform and it requires no software installation when run on the cloud, but it can also be run locally or via Amazon’s EC2 service (Minevich et al., 2012).

### Comparative genomics

Comparative genomics is the study of the relationship of genome structure and function across different biological species or strains. Large comparative genomics studies and tools are becoming increasingly more compute-expensive as the number of available genome sequences continues to rise. The capacity and cost of local computing infrastructures are likely to become prohibitive with the increase, especially as the breadth of questions continues to rise. Alternative computing architectures, in particular cloud computing environments, may help alleviate this increasing pressure and enable fast, large-scale, and cost-effective comparative genomics strategies going forward. Cloud computing services have emerged as a cost-effective alternative for cluster systems as the number of genomes and required computation power to analyze them increased in recent years (Wall et al., 2010). The tsunami of DNA data brought by the next-generation and third generation sequencing techniques is demanding computationally intense application for simulation and processing of data which cannot be brought by the traditional bioinformatic tool analysis. One of the intensive computation demands is Reciprocal Shortest Distance (RSD) algorithm for comparative genomics which further increases with the increase in genome size to be analyzed. Wheat, having a very large genome as compared to rice, corn, and even human, having >80% repetitive DNA and tangled ancestry (derived from three different grass families) makes it unsuitable for many research purposes. But cloud is a subtle way of resolving this problem without worrying about the genome size and type. Basically, RSD uses three bioinformatics application for comparison purpose (Kudtarkar et al., 2010; Wall et al., 2010). They are:

1. BLAST: Protein sequence from one genome is compared with the other whole-genomes available. The list of hits above a threshold is selected.
2. CLUSTALW: The hits are individually aligned with the original query sequence. Again a set of hits above threshold is selected.
3. Codeml: A program from PAML is used for shortest distance calculation by maximum likelihood estimation of amino acid substitution.

Then using phylogenetic analysis sequence having shortest distance is retained and it is checked by reciprocal blast against genome containing the query sequence. The shortest distance is calculated from hits for original sequence. This is iterated several times for complete studies.

This process involves a long toolchain with several formats for interchange between several tools often also requiring changes in between the output of one tool and the input of the next. Setting up the long tool chain with proper configuration in each system becomes a very tedious process in the traditional approaches of grid computing or distributed computing. In cloud computing, cloud providers provide a simple way to duplicate a cloud system. This way we only need to setup a single instance. This instance can be distributed on multiple servers by duplicating them automatically, thereby making the process easy to control as only one instance had to be setup.

### Genome informatics

Under the traditional flow of genome information, data centers used the internet as the pipeline for raw and simulated sequence information. The internet also provided users with direct or indirect access (i.e., through third-party webpages). Problems arose for power users, who needed to maintain their own computation and storage clusters and also preserve local copies of sequence datasets. Similar problems affected organizations maintaining websites and value added integrators.

Nowadays, one can establish an account with Amazon Web Services (the earliest service provider to realize a practical cloud computing environment) or one of the other commercial vendors, launch a virtual machine instance from a wide variety of generic and bioinformatics-oriented images and attach any one of several large public genome-oriented datasets. For virtual machine images, one can choose images pre-populated with Galaxy: a powerful web-based system for performing many common genome analysis tasks, Bioconductor: a programming environment that is integrated with the R statistics package, GBrowse: a genome browser, BioPerl: a comprehensive set of bioinformatics modules written in the Perl programming language, JCVI Cloud BioLinux: a collection of bioinformatics tools including the Celera Assembler, and a variety of others. Several images that run specialized instances of the UCSC Genome Browser are under development. The biggest obstacle to moving to the cloud may well be network bandwidth. A typical research institution will have network bandwidth of about a gigabit/second (roughly 125 MBps). Cloud computing is an attractive technology at this critical juncture. This way now power users can create on demand virtual compute clusters which have a direct access to the datasets and need not to download and save local copies to their system (Stein, 2010).

Another such platform is the Windows-based cloud computing platform, Microsoft Azure, which is not yet as frequently used by the research community as Amazon Web Services. If users aim to transfer their Windows-based applications such as those coded in ASP.NET into a cloud space, Azure can be one of the best candidates with the least modification of the current implementation (Kim et al., 2012).

### Metagenomics

Metagenomics generally refers to the creation of general catalogs of the genomic constituents of organisms in natural or engineered environments, typically based on whole-genome sequencing of DNA extracted from highly mixed populations. Metagenomics is a relatively new technique that allows the analysis of DNA samples taken from a variety of environments: marine, terrestrial, and so forth. Meta Genome Rapid Annotation using Subsystem Technology (MG-RAST; Meyer et al., 2008) is currently the leading metagenomics analysis facility. It is growing quickly, with 700 new datasets added between January and April 2009 alone. Many of these datasets stem from previous-generation DNA sequencing technology and contain on average only 100 Mbp of data.

Meta Genome Rapid Annotation using Subsystem Technology has a simple workflow; first, the data in fasta format is chunked into smaller pieces, then each chunk using BLAST is searched for similarities within the database (Wilkening et al., 2009). This BLAST part is heavily computation demanding and thus parallel computation is needed along with some hardware development. This can be done faster with cloud computing. The developed hardware and required environment is provided by provider and the computation is done in parallel over various distributed servers to save time and cost. But the scenario changed here and from the user point of view, cloud computing was costlier than native approach (Qiu et al., 2010).

Estimating metagenomic taxonomic content constitutes a key problem in metagenomic sequencing data analysis and extracting such content from high-throughput data of next-generation sequencing is very time-consuming. CloudLCA is a parallel LCA algorithm that significantly improves the efficiency of determining taxonomic composition in metagenomic data analysis. In comparison with MEGAN, a well-known metagenome analyzer, the speed of CloudLCA is up to five more times faster, and its peak memory usage is approximately 18.5% that of MEGAN, running on a fat node. CloudLCA can be run on one multiprocessor node or a cluster. CloudLCA is a universal solution for finding the lowest common ancestor, and it can be applied in other fields requiring an LCA algorithm (Zhao et al., 2012). Another such tool is the CloVR-metagenomics pipeline which employs several well-known tools and protocols for the analysis of metagenomic whole-genome shotgun (WGS) sequence datasets.

## Advantages of Cloud Computing

1. Elasticity: Cloud computing maintains exceptional granularity on usage of services as a function of time. By this we mean it not only identifies specific combinations of applications for use with an individual service, but it also actively releases these applications when not in use by the individual. This greatly increases efficiencies, which results in reduced costs to the end-user. Similar improvements in efficiencies are associated with implementing updates to these individual applications.
2. Cost-effectiveness: From the users’ point of view, cost-effectiveness can be examined under the following sub-headings:
  (a) Hardware acquisition, installation, and maintenance: paying for virtual hardware on demand is much cheaper than buying and maintaining equipment that is constantly being improved in performance. In this respect, various hidden costs need to be considered, including amortization of capital associated with equipment acquisition and also installation (for example, dedicated clean environments, and air conditioning).
  (b) Software Costs: Generally, software licenses are costlier when invested in computers at every node, rather than being installed on service provider and serving node computers virtually.
  (c) Server Costs: Although server hardware rates are going down rapidly since on demand virtual hiring is much cheaper. This is because of the fact that in virtual hiring, one pays a very low rate for the compute capacity one actually consumes. The following three instances describe the advantages of virtual hiring in much detail as incorporated in Amazon EC2:
    - On-demand instances – On-demand instances lets you pay for compute capacity by the hour with no long-term commitments. This frees you from the costs and complexities of planning, purchasing, and maintaining hardware and transforms what are commonly large fixed costs into much smaller variable costs.
    - Reserved Instances – Reserved Instances give you the option to make a low, one-time payment for each instance you want to reserve and in turn receive a significant discount on the hourly charge for that instance. There are three Reserved Instance types (Light, Medium, and Heavy Utilization Reserved Instances) that enable you to balance the amount you pay upfront with your effective hourly price.
    - Spot Instances – Spot Instances allow customers to bid on unused capacity and run those instances for as long as their bid exceeds the current Spot Price. The Spot Price changes periodically based on supply and demand, and customers whose bids meet or exceed it gain access to the available Spot Instances. If you have flexibility in when your applications can run, Spot Instances can significantly lower your Amazon EC2 costs.
3. Reliability and Security: In cloud computing, since data is stored virtually, typically between multiple physical locations, the data is more secured in terms of physical events [a user’s system crash, environmental disruptions (earthquake, floods, etc.) and physical theft]. This happens to be more secure in regard to data loss since there are multiple backups in different locations.

## Challenges of Cloud Computing

Despite the benefits outlined above, cloud computing faces many challenges which need to be overcome in order to fully exploit these benefits and to continue to improve its capabilities. Qian et al. (2009) have described some of these challenges:

1. Data Security: Since data is maintained, served, and secured by the service providers, the end-user has to rely on the provider for data security. This is a very big issue which is hindering commercialization of the cloud application. For example there has been a case of private user data being stolen across Dropbox accounts due to lack of security measures by the provider.
2. Data Recovery and Management Systems: In situations where any cloud provider becomes underserved due to any reason, the resultant damage could be severe, and cannot be rectified in situations where data is lost. So more secured and safe recovery systems are required that are completely reliable. There have been recent cases such as the situation with the take-down of sites like Megaupload.com due to legal copyright issues. In the case of megaupload lots of data belonging to users not affected by the copyright cases were still lost forever. A similar such situation could affect our data stored on the cloud if the cloud provider was to be taken down. In order to recover from such a scenario one might consider replicating the results of the simulation after being processed on the cloud across several cloud providers so as to provide a means to recover.
3. Bioinformatics and Computational Biology Problems: Multiple areas of biology continue to be introduced requiring computational and bioinformatics problem-solving techniques (Galbraith, 2011). A major problem in this field is epistemological (Mushegian, 2011): there is a misapprehension that dry lab results are less accurate than wet lab results which has confined its use in research work.
4. Metadata Management and Cloud Provenance: Data provenance focuses on data flows, data history, inputs, outputs, and data transformations that occur in a cloud. This further complicates the management of metadata, since documents are no longer stored on local machines or file servers, but instead stored remotely at third-party data centers. The problem will be complicated by the fact that users can modify documents on their third-party data centers and transmit them directly without sending them via a security solution that automatically checks for metadata and/or scrubs it before documents are sent.
5. Server solutions now include new metadata removal options that protect the entire organization by scrubbing email attachments transmitted via mobile devices and corporate web mail. The downside is that server solutions give end-users less control over their documents, but they may still be the preferred solution for environments where end-user control is not a priority.

These are the major areas to be considered recently to bring the organization and users on the virtual environment. There are many technical obstacles for adoption, growth, policy, and business obstacles for shifting to cloud computing.

## Conclusion

Cloud computing is a field which is combined deployment of many ideas, technologies from different subject areas. Thus it is overall implementation of applications to handle the real world problem, the tsunami of data. There is a lot more opportunities in the fields of research and development as it is a growing field and yet to be fully commercialized. The cloud computing in biological system will change the scenario of the approach toward solution of biological problems with much faster data acquisition and analysis rates. It may be deployed for development of DNA identifiers based on genome sequences as technology advances. There have been a lot of applications of cloud computing in the last few years in the field of genomics and other biological research and development sector. New cloud computing tools and algorithms have been developed and successfully implemented to manage the huge data and to analyze them more efficiently in much lesser time.

## Conflict of Interest Statement

The authors declare that the research was conducted in the absence of any commercial or financial relationships that could be construed as a potential conflict of interest.
